# Maternal Supplementation of Vitamin E or Its Combination with Hydroxytyrosol Increases the Gut Health and Short Chain Fatty Acids of Piglets at Weaning

**DOI:** 10.3390/antiox12091761

**Published:** 2023-09-13

**Authors:** Hernan D. Laviano, Gerardo Gómez, Rosa Escudero, Yolanda Nuñez, Juan M. García-Casco, María Muñoz, Ana Heras-Molina, Clemente López-Bote, Antonio González-Bulnes, Cristina Óvilo, Ana I. Rey

**Affiliations:** 1 Departamento Producción Animal, Facultad de Veterinaria, Universidad Complutense de Madrid, Avda. Puerta de Hierro s/n., 28040 Madrid, Spain; 2 Instituto Regional de Investigación y Desarrollo Agroalimentario y Forestal de Castilla-La Mancha (IRIAF), 13700 Tomelloso, Spain; 3 Departamento de Mejora Genética Animal, Instituto Nacional de Investigación y Tecnología Agraria y Alimentaria, INIA, CSIC, Ctra Coruña km 7.5, 28040 Madrid, Spain; 4 Departamento de Producción y Sanidad Animal, Facultad de Veterinaria, Universidad Cardenal Herrera—CEU, CEU Universities, C/Tirant lo Blanc, 7, Alfara del Patriarca, 46115 Valencia, Spain

**Keywords:** vitamin E, hydroxytyrosol, piglet’s SCFAs, gut health, sow diet, antioxidants

## Abstract

An adequate intestinal environment before weaning may contribute to diarrhea predisposition and piglet development. This study evaluates how the dietary supplementation of vitamin E (VE) (100 mg/kg), hydroxytyrosol (HXT) (1.5 mg/kg) or the combined administration (VE + HXT) given to Iberian sows from gestation affects the piglet’s faecal characteristics, short chain fatty acids (SCFAs), fatty acid profile or intestinal morphology as indicators of gut health; and quantify the contribution of the oxidative status and colostrum/milk composition to the piglet’s SCFAs content and intestinal health. Dietary VE increased isobutyric acid (iC4), butyric acid (C4), isovaleric acid (iC5), and ∑SCFAs, whereas HXT increased iC4 and tended to decrease ∑SCFAs of faeces. Piglets from HXT-supplemented sows also tended to have higher faecal C20:4n-6/C20:2 ratio C22:6 proportion and showed lower *occludin* gene expression in the duodenum. The combination of both antioxidants had a positive effect on iC4 and iC5 levels. Correlation analyses and regression equations indicate that faecal SCFAs were related to oxidative status (mainly plasma VE) and colostrum and milk composition (mainly C20:2, C20:3, C20:4 n-6). This study would confirm the superiority of VE over HXT supplementation to improve intestinal homeostasis, gut health, and, consequently piglet growth.

## 1. Introduction

Maintaining good intestinal health is increasingly important in today’s intensive animal production. Adequate development of the intestinal epithelium, integrity of the mucosa, and intestinal barrier that allow the maintenance of homeostasis and the correct physiological functionality at this level guarantee efficient use of nutrients and an adequate immune response by the host [1]. However, stressful situations can increase lipid peroxidation of biomembranes, promoting changes in structure, fluidity, and wall permeability, leaving the cell unprotected against external agents such as pathogenic microorganisms and toxins [2]. Thus, during weaning (one of the most critical and stressful moments in the life of the piglet), the still immature intestinal epithelium has to face the colonization of new microorganisms, the presence of new solid foods, and an increased oxidative stress, producing changes in digestive and immune function that can trigger digestive disorders and a reduction in nutrient absorption that adversely influence normal growth [1].

Many dietary interventions and selected feed additives have been tested in the last years in order to prevent this digestive dysfunction by enhancing the immune response, stimulating digestive function, increasing the gut barrier integrity [3], or establishing beneficial gut microbes [4]. Moreover, it has been observed that intestinal health is highly influenced by certain nutrients present in the food that, due to their fermentation, favor the growth of beneficial microbial populations and the production of derived metabolites, such as short-chain fatty acids (SCFAs) [5,6,7]. Gut microbiota-derived-SCFAs are being widely investigated since they appear to act not only as an interesting energy source for colonocytes [8] but also as mediators in the activation of various functions in the body. They are essential for reducing local inflammation, protecting against pathogen infiltration, and maintaining intestinal barrier integrity [9]. In addition, SCFAs have been found to be involved in glycemic control and appetite regulation [10] and may play a role in the developmental programming of obesity [11].

Antioxidant compounds have also been shown to be effective in reducing intestinal free radicals under stressful conditions, helping maintain the intestinal mucosa, and modulating gut microbial communities [2]. Thus, vitamin E (VE) has shown protective effects on intestinal barrier function by their radical scavenger properties at the membrane level [12] and upregulated expression of tight junction proteins in healthy piglets [13]. Their different isoforms (alpha- and gamma-tocopherol) also seem to be effective in mitigating intestinal disorder by protecting the gut barrier integrity and favorable changes of gut microbiota in colitis-induced mice [14]. Also, in vitro VE can induce a pronounced effect on microbiota metabolism end-products such as SCFAs [15].

Polyphenol compounds have also been shown to induce metabolic changes when interacting with microbial enzymes in the gut [2]. One of the strongest antioxidant polyphenols is hydroxytyrosol (HXT) [16], obtained from olive derivatives such as leaves, fruit, oil, or oil production waste products, and it naturally originated during the hydrolysis of the secoiridoid compound oleuropein [16] during processing and storage. Thus, HXT promoted the regeneration of the intestinal barrier and maintained intestinal functional homeostasis by increasing the regeneration of globlet cells and the expression of mucid protein and tight junction proteins in rats with induced colitis [17]. Moreover, it has been reported to have anti-inflammatory effects on induced ulcerative colitis in rats by enhancing colonic antioxidant capacities [18,19] and modulating gut microbiota.

Despite the positive effects of VE and HXT on intestinal health in laboratory animals under induced digestive disorders, there is a lack of information on the comparative effect of these two antioxidants in weaned piglets. There is also a lack of information on the effect that the administration of these antioxidants to the sow may have on the SCFA transmission or piglet intestinal production or whether the combined use of both antioxidants could be more beneficial than the independent administration of each of the compounds on gut health. In addition, in a previous paper, Laviano et al. [20] reported that both (VE or HXT) given to Iberian sow produced changes in the composition (mainly fatty acid profile) of colostrum and milk, and this resulted in different lipid stability and oxidative status of the piglet. However, this previous study did not address how these changes in feed during lactation might affect the piglet’s gut health by changing the faecal SCFA levels or intestinal structure and morphology.

It is hypothesized that dietary antioxidant administration to the sows, as well as the concomitant changes in colostrum and milk induced by either VE or HXT supplementation, might modify the intestine membrane structure, faecal SCFAs concentrations, and piglet’s gut health in a different way.

Therefore, the aim of the present study was to evaluate how the dietary supplementation of vitamin E (VE) (100 mg/kg), hydroxytyrosol (HXT) (1.5 mg/kg), or the combined administration (VE + HXT) given to Iberian sows from day 85 of gestation affects the characteristics of the stool, SCFAs composition, or faecal fatty acid profile of the offspring as indicators of gut health; and secondly, to quantify the contribution of the feeding (colostrum or milk) or the piglets oxidative status to the gut SCFAs profile; and the relationship between faeces composition and piglets growth pattern.

## 2. Materials and Methods

### 2.1. Chemicals

All analytical grade chemicals were supplied by the following companies: Sigma-Aldrich (Alcobendas, Madrid, Spain), Panreac (Castellar del Vallès, Barcelona, Spain), or Scharlau (Sentmenat, Barcelona, Spain).

### 2.2. Ethics Statement

Experimental procedures (carried out at the animal facilities of Dehesón del Encinar, Oropesa, Toledo, Spain),were in compliance with the Spanish Policy for Animal Protection (RD53/2013) [21] and European Union Directive 2010/63/UE [22] for the care and use of animals in research. The experimental procedures were approved by the INIA Committee of Ethics in Animal Research (report ORCEEA 2019-10). 

### 2.3. Animals, Experimental Procedures and Diets

Iberian sows (n = 50; half primiparous and half multiparous between 4–5 parity) with an average body weight of 107.2 kg ± 29.8 were pregnant by natural service (Dehesón del Encinar, Oropesa, Toledo, Spain). During the pre-experimental period, sows were given a standard grain-based diet (g/kg: 888 dry matter, 124.6 protein, 29.9 fat, 49.3 fiber, 62.1 ash; and 3050 kcal/kg Metabolizable Energy) (Sanchez Romero Carvajal, Spain). At day 85 of pregnancy (126.2 kg ± 29.3), the sows were divided into four experimental groups (12–13 per dietary treatment with equal distribution of primiparous and multiparous) and started receiving four different experimental diets until weaning (28 days after delivery). The experimental diets (Table A1) were: (1) control group; 30 mg of α-tocopheryl acetate/kg feed; (2) VE group: 100 mg of α-tocopheryl acetate/kg feed; (3) HXT group: 30 mg α-tocopheryl acetate/kg feed and 1.5 mg hydroxytyrosol/kg feed and (4) VE+HXT: 100 mg/kg of α-tocopheryl acetate/kg feed + 1.5 mg hydroxytyrosol/kg feed. Feed administration was adjusted to fulfill daily maintenance requirements [23]. The dose of 30 mg α-tocopheryl acetate/kg feed was used for basal diet supplementation to reach the α-tocopherol levels recommended by the NRC [23] for sows and was obtained from DSM Nutritional Products (Alcalá de Henares, Madrid, Spain). The hydroxytyrosol extract (Olea europaea L. dry extract, N20130102) was obtained from Natac (Alcorcón, Madrid, Spain) and was certified by the company to contain a minimum of 1.5% hydroxytyrosol.

After farrowing, live piglets (averaged litter size of 7–8 per treatment) were intramuscular injected with iron behind the ear (Previron 200, Hipra, Talavera de la Reina, Toledo, Spain) within the next 24 h to avoid anemia. Piglets were also tagged with electronic ear tag (MPig Data, Madrid, Spain), tail was cut, and males were castrated in accordance with RD 53/2013 [21]. Piglets stayed with their mother until weaning.

### 2.4. Sample Collection

Colostrum milk sampling in sows was described in a previous paper [20]. Briefly, colostrum and milk samples (n = 7 per treatment obtained from sows with similar litter size) were taken after farrowing, and at days 7 and 20 by hand-milking, were put into plastic tubes, kept into a refrigerator, and then frozen until analysis.

Blood samples from sows were taken at day 110 of gestation and day 20 of lactation (n = 7 per dietary treatment). For blood sampling in piglets (day 20 of lactation), at least one male piglet (n = 7–8 per treatment) was chosen at random from the same mother from which blood was obtained. Blood samples were taken in sterile EDTA tubes (Vacutainer, BD, Franklin Lakes, NJ, USA), immediately centrifuged (2500 rpm for 10 min) to obtain plasma, and then transported in dry ice and kept at −80 °C until analysis (less than 1 month).

Five days after weaning (day 33), 48 male piglets (n = 12 per dietary treatment; half from multiparous and half from primiparous sows) were selected, taking into account those males previously used for sampling and additional ones were chosen to obtain at least 1 male piglet per litter (6.1 kg ± 1.3) and euthanized in compliance with RD 53/2013 [21]. Then, blood was taken, and plasma was obtained as described above. In addition, faecal samples were collected directly from the last section of the large intestine (colon after the caecum) and placed inside a 10 mL plastic container. Samples were transported in dry ice and kept at −80 °C until analysis (less than 1 month). Moreover, a small portion of the intestine (duodenum) was taken for RNA expression analysis and immediately frozen in liquid nitrogen. Samples of the small intestine (duodenum) were also taken for morphology study and fixed in 10% phosphate-buffered formalin (Fisher Scientific, Madrid, Spain) in plastic tubes for evaluation of the villus height, crypt depth, number of villi, number of crypts, and number of goblet cells (Micros Veterinaria, León, Spain).

### 2.5. Laboratory Analysis

#### 2.5.1. Antioxidant’s Enzyme Analysis in Plasma

Analysis of the concentration of superoxide dismutase (SOD), catalase, and total glutathione (GSH) of piglet’s plasma was carried out spectrophotometrically (Multiscan ScanGo, Thermo-Fisher Scientific, Alcobendas, Spain) by means of commercial kits (Arbor Assays, Ann Arbor, MI, USA) according to manufacturer’s instructions. SOD and catalase were expressed as U/mL, whereas GSH was calculated as µM.

#### 2.5.2. Moisture, Fat Content, and Fatty Acid Profile of Faecal Samples

Moisture of faecal samples was quantified gravimetrically during the lyophilization process [24]. The empty plastic container was weighed before faecal sample collection and later contained the sample after the lyophilization (Lyoquest, Telstar, Tarrasa, Spain) until constant weight. Moisture was calculated by the difference between the initial and final weights and expressed as a percentage.

Faecal total lipids were extracted as described elsewhere [24] and then analysed for fatty acid profile composition. Freeze-dried samples (Lyoquest, Telstar, Tarrasa, Spain) were weighted in an Eppendorf, and a solvent mixture of dichloromethane–methanol 8:2 was added. After homogenisation in a mixer mill (MM400, Retsch technology, Haan, Germany) and centrifugation during 8 min at 10,000 rpm (Hermle Z383-K; Wehingen, Germany), the upper layer containing lipids were collected. The lipid content was quantified gravimetrically after evaporation of the solvent in a nitrogen stream. Fatty acid methyl esters (FAMEs) were obtained by the addition of methanol/toluene/H_2_SO_4_ (88:10:2 by volume) after heating the lipids (80 °C for 1 h) [25]. Then, FAMEs were extracted with hexane and separated after sample injection in a gas chromatograph (HP 6890 Series GC System; Hewlett Packard, Avondale, PA, USA). The gas chromatograph was provided with an automatic injector, a capillary column (HP-Innowax polyethylene glycol, 30 m × 0.316 mm × 0.25 µm), and a flame ionization detector (held at 250 °C). After injection, the oven temperature was increased from 170 °C to 210 °C at a rate of 3.5 °C/min, then to 250 °C at a rate of 7 °C/min [13,24]. Identification and quantification of the FAMEs were made by comparing the retention times with those of authentic standards (Sigma-Aldrich, Alcobendas, Spain). Results were expressed as grams per 100 g of quantified fatty acids.

The desaturase and elongase (ratio of C14:0 to C16:0, C16:0 to C18:0, and C18:0 to C20:0) indices were calculated as described elsewhere [20].

#### 2.5.3. Analysis of Short-Chain Fatty Acids in Faecal Samples

Determination of short-chain fatty acids in the faecal samples was carried out as described by Higueras et al. [24]. Briefly, freeze-dried stool samples were accurately weighed, and distilled water was added to obtain an approximated average dispersion of faeces in water of 17%. Then, samples were homogenised for 5 min at 30 Hz in a Mixer Mill MM400 (Retsch technology, Haan, Germany) and centrifuged for 10 min. at 10,000 rpm (Hermle Z383-K; Wehingen, Germany) to obtain the superior phase that was transferred into a vial where the internal standard (20 mM 4-methylvaleric acid solution) was spiked. Phosphoric acid (25%) was added to adjust the pH to 2–3. The extraction was repeated three times, and then this solution was placed in vials for gas chromatography injection. Chromatographic analysis was carried out using an Agilent 6850N GC system equipped with a fused-silica capillary column (DB-FFAP 125-3237, 30 m × 0.53 mm i.d. coated with a 0.50 µm thickness film) (Agilent Technologies Inc., Santa Clara, CA, USA), an automatic injector and a flame ionisation detector (FID) (Agilent Technologies, Waldbronn, Germany). The carrier gas was nitrogen at a constant pressure of 15 psi. The initial oven temperature was 100 °C maintained for 0.5 min, raised to 180 °C at 8 °C/min and held for 1.0 min, then increased to 200 °C at 20 °C/min and finally held at 200 °C for 5 min [24]. The temperatures of the FID and the injection port were 240 °C and 200 °C, respectively. Data handling was carried out with HP ChemStation Plus software (Agilent Technologies, Waldbronn, Germany). Identification and quantification were carried out using pure standards (Sigma Aldrich, Alcobendas, Spain). An aqueous stock standard solution was prepared for each acid with a concentration of 400 mM for acetic (C2), propionic (C3), and n-butyric acid (C4); 200 mM for n-valeric (C5) and i-valeric acid (iC5); 100 mM for i-butyric acid (iC4); 50 mM for n-caproic acid (C6).

#### 2.5.4. Tocopherol Quantification in Faecal Samples

The Vitamin E (α-tocopherol) concentration in faecal material from piglets was extracted by direct extraction as described by Rey et al. [26] with little modifications. Briefly, different volumes of ethanol were added to duplicate stool samples (0.25 mg), followed by homogeneization (5 min at 30 Hz in a Mixer Mill MM400) to a final volume of 2 mL. Tocopherol was extracted by centrifugation at 10,000 rpm for 5 min at 4 °C (Hermle Z383-K; Wehingen, Germany), and the supernatant was collected and evaporated by N_2_ stream. The remaining residue was dissolved in ethanol and injected into an HPLC (HP 1200, equipped with a diode array detector and a reverse RP-18 column) (Agilent Technologies, Waldbronn, Germany) [27]. Identification and quantification were carried out using the pure compound (Sigma-Aldrich, Alcobendas, Madrid, Spain). Results were expressed as µg of α-tocopherol per g of faeces.

#### 2.5.5. Gene Expression Analysis in Intestine Samples

Total RNA was obtained from duodenum samples (50–100 mg of the whole intestinal wall) from the 48 piglets sacrificed 5 days after weaning, using the RiboPure^TM^ RNA isolation kit (Ambion, Austin, TX, USA), following the manufacturer’s recommendations. The obtained RNA was quantified using NanoDrop equipment (NanoDrop Technologies, Wilmington, DE, USA), and the RNA quality was assessed with an Agilent 2100 bioanalyzer device (Agilent Technologies, Palo Alto, CA, USA). The RNA obtained was retrotranscribed using Superscript II (Invitrogen Life Technologies, Paisley, UK) and random hexamers, following the supplier’s instructions, and employed for the quantification of the expression of zonula occludens 1 (*ZO1*) and occludin (*OCLN*) genes, following the procedure described in Benitez et al. [28]. Primer pairs were designed using Primer Select software (DNASTAR, Madison, WI, USA) from the available GENBANK and/or ENSEMBL sequences. Primer pairs covered different exons to assure the amplification of the cDNA. Information on primer sequences is available in Table A2. The most stable endogenous genes out of *GAPDH*, *ACTB*, *TBP*, *PPIA*, *EEF2*, and *B2M* were selected for data normalization after the evaluation of their stability with the Genorm software [29]. The *GADPH* and *EEF2* genes were selected as the most stable endogenous genes. Normalization of gene expression data was performed by dividing by the normalization factor calculated from the expression of the two reference genes using Genorm software.

#### 2.5.6. Intestinal Morphology

For the histological study, samples of the duodenum were placed in histological cassettes, and their inclusion in paraffin was carried out by passage through alcohols of increasing concentration, xylene, and molten paraffin. Once the histological blocks were obtained, two 2.5 µm thick sections were made, which were stained with hematoxylin-eosin and PAS (Periodic Acid-Schiff) staining. In the preparations stained with hematoxylin-eosin, microphotographs were obtained with a Leica ICC50W™ camera (Dismed, Gijón, Spain) coupled to a Leica DM1000™ microscope (Dismed, Gijón, Spain). Using the Image JTM software (version 1.53, National Institutes of Health, Bethesda, MD, USA, [30]), 10 measurements of complete villous length and complete crypt depth were made. Villus height was measured from the tip to the villus-crypt junction, and crypt depth was measured from the crypt mouth to the base. In addition, the number of villi and crypts were counted in 3 segments of at least 1 mm in length. In the preparations stained with the PAS stain, the number of total goblet cells was counted in 10 villi for each animal.

### 2.6. Statistical Analysis

For analysis of data, the general linear model (GLM) procedure contained in SAS (version 9.4; SAS Inst. Inc., Cary, NC, USA) was used as random and included the fixed effects of VE or HXT with their interaction in a full factorial model. The individual piglet was considered the experimental unit. Comparison between means was conducted using the post-hoc Duncan test. Data are presented as the mean of each group, and root mean square error (RMSE) together with the significance levels (*p* value). Pearson correlation coefficients between faecal SCFAs and faecal characteristics, faecal SCFAs and piglets/sows oxidative status, faecal SCFAs and colostrum/ milk composition (analysed in Laviano et al. [20]), faecal SCFAs and fatty acids of faeces or between SCFAs and piglet’s body size (measured in a previous paper; Gómez et al. [31]), were carried out using Statgraphics-19 program (Statgraphics Technologies, The Plains, VA, USA). Correlations between piglets’ intestinal morphology and sows or piglets’ oxidative status and between piglets’ intestinal morphology or colostrum and milk composition were also evaluated by Statgraphics-19 program. Regression equations (between those parameters in which correlations were significant) were used to evaluate the linear response (Statgraphics-19, Statgraphics Technologies, The Plains, VA, USA).

Differences were considered statistically significant when *p* < 0.05, whereas *p* > 0.05 and <0.1 was considered as a trend.

## 3. Results

### 3.1. Oxidative Status of Weaned Piglets

**Figure 1:**
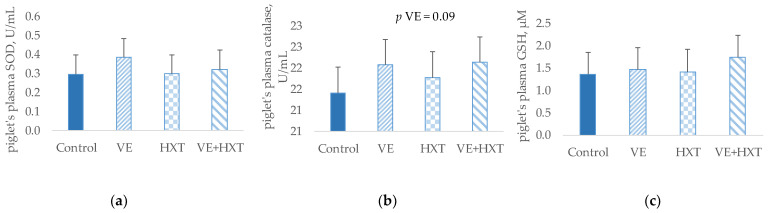
Antioxidant enzymes concentrations (**a**) superoxide dismutase (SOD); (**b**) catalase; and (**c**) total glutathione (GSH) in piglet’s plasma (post-weaning) from sows given α-tocopheryl acetate (VE: 30 vs. 100 mg/kg) or hydroxytyrosol (HXT: 0 vs. 1.5 mg/kg) during gestation and lactation. Control = 30 mg of α-tocopheryl acetate/kg feed + 0 mg/kg hydroxytyrosol; VE = 100 mg of α-tocopheryl acetate/kg feed + 0 mg/kg hydroxytyrosol; HXT = 30 mg of α-tocopheryl acetate/kg feed + 1.5 mg/kg hydroxytyrosol; VE + HXT = 100 mg of α-tocopheryl acetate/kg feed + 1.5 mg/kg hydroxytyrosol.

The oxidative status of weaned piglets measured as the quantification of the concentration of antioxidant enzymes SOD, catalase, and GSH in plasma samples is presented in Figure 1.

Supplementation of sows diets with VE produced a tendency to increase plasma catalase concentration (*p* = 0.09) when compared with the non-supplemented groups; however, no significant changes were observed for the other antioxidant enzymes. No significant changes were observed in the piglet’s plasma antioxidant enzymes by the sow’s HXT supplementation, nor was a significant interaction observed by the administration of both antioxidants on the concentration of piglet antioxidant enzymes.

### 3.2. Faeces Characteristics

The characteristics of piglet faeces from sows given VE or HXT are presented in Table 1. Moisture, fat content, or VE concentration of faeces were not statistically modified by the dietary treatment (VE or HXT) of their mothers. Moreover, no interaction effects were observed, and the supplementation of both antioxidants to the sow’s diets did not change the characteristics of the piglet’s faeces.

### 3.3. Short-Chain Fatty Acid (SCFAs) Composition of the Piglet’s Faeces

The short-chain fatty acid content (SCFAs) of piglet faeces changed according to the sow’s dietary supplementation (Table 2).

The more marked effects were observed by the VE administration that increased the total SCFAs of piglet’s faeces (*p* = 0.022). VE supplementation also improved the specific production of isobutyric acid (iC4) (*p* = 0.007), isovaleric acid (iC5) (*p* = 0.003), and butyric acid (C4) (*p* = 0.050) and a tendency to have greater content of valeric acid (C5) (*p* = 0.079) was observed. However, VE supplementation did not affect the content of acetic acid (C2), propionic acid (C3), or caproic acid (C6).

On the other hand, HXT supplementation tended to increase iC5 (*p* = 0.090), but it decreased C4 (*p* = 0.018) and total SCFAs (*p* = 0.022) in piglet’s faeces. The content of C2, C3, iC4, C5, or C6 of piglet’s faeces did not change by the administration of 1.5 mg/kg HXT to the sows.

The combination of both antioxidants in the sows resulted in an increase of piglet’s faecal iC4 (*p* = 0.017) and iC5 (*p* = 0.012) when compared to the single administration of VE or HXT (interaction effect). However, the combination of both antioxidants did not significantly affect (*p* > 0.050) on the total faecal SCFA content when compared to the independent administration.

### 3.4. Relationship between the Piglet’s Oxidative Status and SCFAs

The Pearson correlation coefficients and significant relationships between piglet’s faecal SCFAs and sows’ or piglets’ oxidative status (post-weaning) are presented in Table 3.

Piglets’ faecal ∑SCFAs, iC4 and iC5, and C3, were highly correlated with weaned piglets’ oxidative status. Thus, positive significant relationships were observed for C3 and plasma SOD (r = 0.44; R^2^ = 0.19; *p* = 0.022) or GSH (r = 0.42; R^2^ = 0.18; *p* = 0.033). The highest relationships were observed between iC4, iC5, and piglet’s plasma α-tocopherol, which were positively related (r = 0.70, R^2^ = 0.49; *p* = 0.000; and r = 0.699, R^2^ = 0.47; *p* = 0.000, respectively). The piglet’s plasma α-tocopherol was quantified in a previous paper of the same research project [31] and was higher in those weaned piglets from VE-supplemented sows compared with non-supplemented (1.051 vs. 1.604 µg/mL).

Moreover, the total SCFAs were also directly correlated with the piglet’s plasma VE concentration. The other SCFAs, C2, C4, C5, or C6, did not correlate with any of the parameters related to the oxidative status of piglets. However, the relationship between C5 and C3 and the oxidative status of the sow (data analysed and presented in Gómez et al. [31]) measured as the ratio reduced/oxidized glutathione (GSH/GSSG) was found (r = 0.51; R^2^ = 0.25; *p* = 0.005; and r = 0.45; R^2^ = 0.20; *p* = 0.016, respectively).

### 3.5. Relationship between Colostrum and Milk Composition and SCFAs

Pearson correlation coefficients and significant regression equations between colostrum composition (analysed in a previous paper by Laviano et al. [20]) and faecal SCFAs of post-weaning piglets are presented in Table 4.

Significant negative linear relationships were observed between faecal C4 and colostrum C18:2 n-6 (r = −0.41, R^2^ = 0.17, *p* = 0.045) and between C4 and PUFA of colostrum (r = −0.41, R^2^ = 0.17, *p* = 0.047). The highest linear adjustments and correlations were found between iC4 or iC5 and colostrum fatty acids. Thus, a positive and linear relationship was observed between iC4 and 20:2 of colostrum (r = 0.62, R^2^ = 0.38, *p* = 0.002) and between iC5 and C20:2 (r = 0.67, R^2^ = 0.44, *p* = 0.001). Lower positive adjustments were observed between iC4 and C20:3 n-6 (r = 0.51, R^2^ = 0.26, *p* = 0.013) and between iC5 and C20:3 n-6 (r = 0.54, R^2^ = 0.29, *p* = 0.008). Similarly, C20:4 n-6 of colostrum was positive and linearly related with iC4 (r = 0.54, R^2^ = 0.29, *p* = 0.008) and iC5 (r = 0.60, R^2^ = 0.36, *p* = 0.003).

Pearson correlation coefficients and regression equations between faecal SCFAs of post-weaning piglets and day-7 or day-20 milk composition (analysed by Laviano et al. [20]) from supplemented sows are presented in Table 5.

The highest number of significant linear relationships and correlation values were observed in 7-day milk samples. Moreover, C5, iC4, and iC5 were the SCFAs that presented the highest relationships with 7-day milk fatty acid composition.

Thus, iC4 was positive and linearly related with C18:1 n-9 (r = 0.53, R^2^ = 0.28, *p* = 0.007), a sum of monounsaturated fatty acids (∑MUFA) (r = 0.57, R^2^ = 0.33, *p* = 0.003) and Δ-9-desaturase (r = 0.52, R^2^ = 0.27, *p* = 0.008). However, a negative and linear relationship was observed between iC4 and the sum of saturated fatty acids (∑SAT) (r = −0.44, R^2^ = 0.20, *p* = 0.034). iC5 was also directly related with MUFA mainly C18:1n-9 (r = 0.52, R^2^ = 0.27, *p* = 0.011). C5 was the most related SCFA to the milk composition. The highest linear relationships were observed between C5 and C20:2 (r = 0.68, R^2^ = 0.46, *p* = 0.0003), and C16:0 (r = −0.65; R^2^ = 0.42, *p* = 0.001). Other MUFA, such as C18:1 n-9 and C20:1, were linearly related to C5. Also, C5 was positive and linearly related to polyunsaturated fatty acids such as C20:3 n-6 (r = 0.59; R^2^ = 0.35, *p* = 0.003) and C20:4 n-6 (r = 0.42; R^2^ = 0.18, *p* = 0.036).

Finally, positive linear adjustments were observed between total SCFAs and MUFA of day-7 milk (Table 5). Therefore, the higher the MUFA content of sow milk, the higher the total SCFAs found in piglet faeces.

Moreover, in day-20 milk (Table 5), C5 was positively correlated to C16:1n-9 (r = 0.45; R^2^ = 0.20, *p* = 0.032) and negatively to n-3 fatty acids (r = −0.54; R^2^ = 0.29, *p* = 0.010).

### 3.6. Fatty Acid Composition of the Piglet’s Faeces and Its Relationship with SCFAs

The total fatty acid profile of piglet’s faeces at 5 days postweaning was also studied.

**Figure 2:**
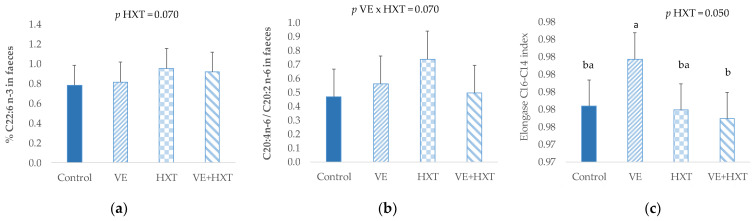
Fatty acid proportion of (**a**) C22:6 n-3; (**b**) ratio C20:4 n-6/C20:2; and (**c**) elongase C14 to C16 in piglet’s faeces from sows given α-tocopheryl acetate (VE: 30 vs. 100 mg/kg) or hydroxytyrosol (HXT: 0 vs. 1.5 mg/kg) during gestation and lactation. Control = 30 mg of α-tocopheryl acetate/kg feed + 0 mg/kg hydroxytyrosol; VE = 100 mg of α-tocopheryl acetate/kg feed + 0 mg/kg hydroxytyrosol; HXT = 30 mg of α-tocopheryl acetate/kg feed + 1.5 mg/kg hydroxytyrosol; VE + HXT = 100 mg of α-tocopheryl acetate/kg feed + 1.5 mg/kg hydroxytyrosol. Letter with different superscript were statistically significant.

No significant changes were observed in the main fatty acid groups (saturated, monounsaturated or polyunsaturated fatty acids) (*p* > 0.05) of piglet’s faeces, and only some punctual fatty acids tended to be affected by antioxidants supplementation to the sow (Figure 2). Thus, the docosahexanoic acid (C22:6 n-3) tended to increase (*p* = 0.070) in piglets faeces from HXT-supplemented sows. Also, the unique administration of HXT to the sows tended to increase the ratio C20:4 n-6/C20:2 (*p* = 0.070) when compared to the combined administration (HXT + VE). Moreover, the elongase C14 to C16 index was lower in piglets from HXT-supplemented sows when compared to non-supplemented (*p* = 0.05).

**Figure 3:**
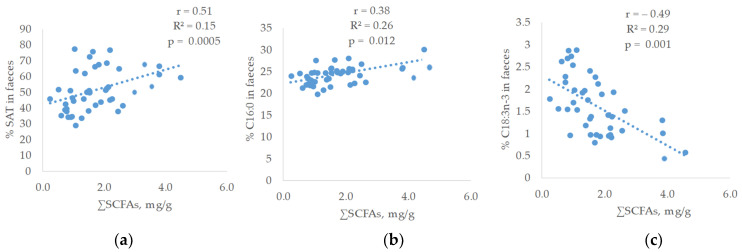

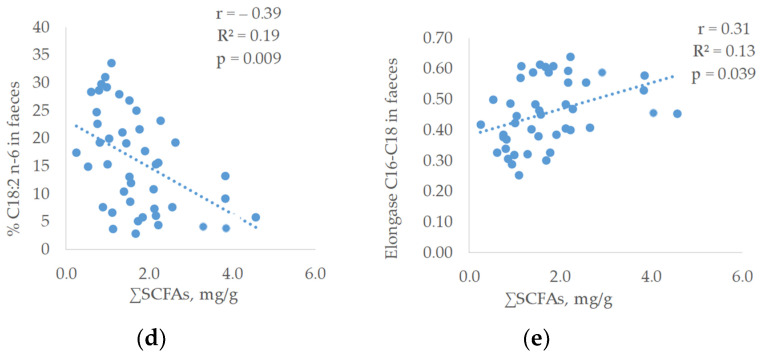
Relationship between ∑SCFAs and (**a**) faecal saturated fatty acids (SAT); (**b**) faecal C16:0; (**c**) faecal C18:3 n-3; (**d**) faecal C18:2 n-6; (**e**) faecal elongase C16-C18 of weaned piglets from sows given α-tocopheryl acetate (VE: 30 vs. 100 mg/kg) or hydroxytyrosol (HXT: 0 vs. 1.5 mg/kg) during gestation and lactation.

Concerning the relationship between the faecal fatty acid proportions and the SCFAs of faeces, linear and significant relationships were observed (Figure 3).

Thus, a linear and positive correlation was found between saturated fatty acids (SAT) (r = 0.51; R^2^ = 0.15; *p* = 0.0005) and SCFAs. The SAT fatty acid that showed the highest relationship was C16:0 (r = 0.38; R^2^ = 0.25; *p* = 0.012). On the contrary, the faecal proportion of polyunsaturated fatty acids was inversely related to SCFAs content of faeces being C18:3 n-3, the fatty acid that reached the highest correlation (r = −0.49; R^2^ = 0.29; *p* = 0.001) followed by C18:2 n-6 (r = −0.39; R^2^ = 0.19; *p* = 0.009). In addition, a linear positive relationship was observed between the total SCFAs and the elongase C16 to C18 (r = 0.31; R^2^ = 0.13; *p* = 0.039).

### 3.7. Relative Expression of Occludin (OCLN) and Zonula Occludens (ZO1) in Intestine Samples

**Figure 4:**
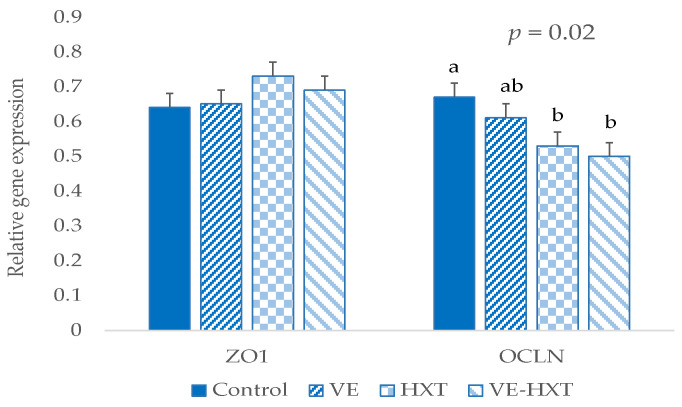
Relative expression of *OCLN* and *ZO1* of weaned piglets from sows given α-tocopheryl acetate (VE: 30 vs. 100 mg/kg) or hydroxytyrosol (HXT: 0 vs. 1.5 mg/kg) during gestation and lactation. Control = 30 mg of α-tocopheryl acetate/kg feed + 0 mg/kg hydroxytyrosol; VE = 100 mg of α-tocopheryl acetate/kg feed + 0 mg/kg hydroxytyrosol; HXT = 30 mg of α-tocopheryl acetate/kg feed + 1.5 mg/kg hydroxytyrosol; VE+HXT = 100 mg of α-tocopheryl acetate/kg feed + 1.5 mg/kg hydroxytyrosol. Letter with different superscript were statistically significant.

No effect of the sow supplementation was found on *ZO1* gene expression in the piglet’s intestine after weaning. However, *OCLN* expression was significantly lower in HXT-supplemented than in control groups (*p* = 0.020, Figure 4).

### 3.8. Intestinal Morphology and Its Relationship with Oxidative Status or Colostrum and Milk Composition

**Figure 5:**
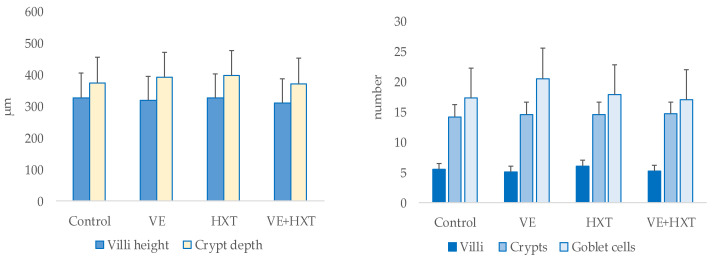
Morphology of piglets duodenum in weaned piglets from sows given α-tocopheryl acetate (VE: 30 vs. 100 mg/kg) or hydroxytyrosol (HXT: 0 vs. 1.5 mg/kg) during late gestation and lactation. Control = 30 mg of α-tocopheryl acetate/kg feed + 0 mg/kg hydroxytyrosol; VE = 100 mg of α-tocopheryl acetate/kg feed + 0 mg/kg hydroxytyrosol; HXT = 30 mg of α-tocopheryl acetate/kg feed + 1.5 mg/kg hydroxytyrosol; VE+HXT = 100 mg of α-tocopheryl acetate/kg feed + 1.5 mg/kg hydroxytyrosol.

The morphology of the piglet’s duodenum (villi height, crypts depth, number of villi, number of crypts, and goblet cells) was not statistically modified by the antioxidant supplementation of their mothers (Figure 5).

There was also no significant interaction observed by the administration of both antioxidants to the mother on the morphology of the duodenum of the piglets.

However, high correlations between some gut histology measurements and the oxidative status of the sow and colostrum or milk composition were observed (Table 6).

Thus, the number of goblet cells was positively related to the sow’s oxidative status measured as plasma oxidized glutathione (GSSG) and malondyaldehyde (MDA) (r = 0.40, R^2^ = 0.16, *p* = 0.041; and r = 0.39, R^2^ = 0.15, *p* = 0.047, respectively). Villi number was also positively correlated with fatty acids of colostrum, mainly C16:1 (r = 0.46, R^2^ = 0.21, *p* = 0.031) and C18:1/C18:0 ratio (r = 0.53, R^2^ = 0.28, *p* = 0.011) and negatively with C18:0 (r = −0.56, R^2^ = 0.32, *p* = 0.007) and SAT (r = −0.49, R^2^ = 0.24, *p* = 0.022). Crypt depth was positively related to ∆5 + ∆6-desaturase activity of colostrum (r = 0.50, R^2^ = 0.25, *p* = 0.017).

Day-20 milk composition also showed a relationship with intestinal morphology. Thus, villi height was negatively and linearly related with C18:2 and n-6 PUFA (r = −0.48, R^2^ = 0.23, *p* = 0.028; r = −0.48, R^2^ = 0.23, *p* = 0.026, respectively). Crypts depth was directly related to α-tocopherol (r = 0.48, R^2^ = 0.23, *p* = 0.027) and C20:1n-9 (r = 0.52, R^2^ = 0.27, *p* = 0.015); and villi number inversely to C18:0 (r = −0.49, R^2^ = 0.25, *p* = 0.026); whereas day-20 milk α-tocopherol correlated negatively with the number of goblet cells (r = −0.48, R^2^ = 0.23, *p* = 0.032).

### 3.9. Relationship between Faecal Composition and Growth Pattern of Piglets

**Figure 6:**
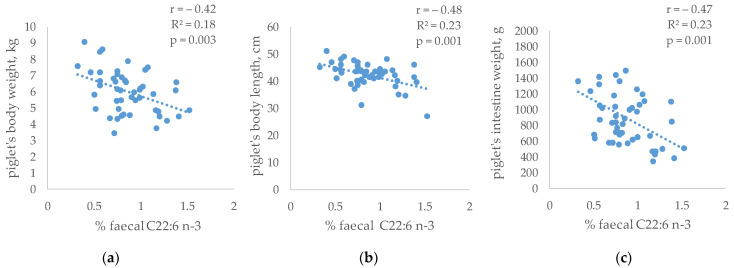
Relationship between faecal C22:6 n-3 and (**a**) piglet’s body weight; (**b**) piglet’s body length; (**c**) piglet’s intestine weight of weaned piglets born from sows given α-tocopheryl acetate (VE: 30 vs. 100 mg/kg) or hydroxytyroxol (HXT: 0 vs. 1.5 mg/kg) from day 85 of gestation (n = 12 per dietary treatment, one per litter).

In addition, the relationship between the faecal composition and the growth pattern of the piglets (measured and published by Gómez et al. [31]) was evaluated (Figure 6). No significant relationships were observed between the SCFAs and the piglet’s growth. However, the piglet’s size was related to the abundance of specific fatty acids in faeces. Thus, faecal C22:6 n-3 was inversely related to body weight (r = −0.42, R^2^ = 0.18, *p* = 0.003), body length (r = −0.48, R^2^ = 0.23, *p* = 0.001) and intestine weight (r = −0.47, R^2^ = 0.23, *p* = 0.001).

## 4. Discussion

Diarrhea after weaning in piglets is a frequent episode in which predisposition depends on the intestinal environment before weaning [32] and could be affected by the type of food, including the presence of certain antioxidants that protect the intestinal membrane. Previous research indicates that milk from mothers supplemented with VE or HXT presented changes in the proportion of fatty acids and concentrations of VE and MDA [20,31]. However, there is no information that quantifies to what extent these changes in milk contribute to end-products of microbiota metabolism and intestinal status, which could have interesting effects on animal health and development.

According to the antioxidant enzyme concentrations measured in the present study, the piglet’s oxidative status was poorly modified by the sow’s antioxidant supplementation. However, additional results obtained in the same research project [31] indicate that piglets from sows given antioxidants had lower plasma MDA concentration as an indicator of oxidative stress control.

Concerning the characteristics of the faeces, these are one of the first determinants of gut health or disease in an animal. Unabsorbed food residues, products of bacterial fermentation, and minerals result in changes in osmolarity, a lack of water reabsorption, and, eventually, diarrhea [33]. Poor absorption of fat and its presence in faeces is another indicator of gastrointestinal disorders [34]. However, neither the humidity nor fat content of faeces was modified in five-day post-weaned piglets according to the sow’s dietary treatment in the present research.

In addition, no differences were found in the concentrations of VE in piglet faeces between the different experimental groups, even though it was properly transferred to the piglets, as indicated by higher plasma concentrations observed in those from VE-supplemented sows [31]. This would indicate that the dose administered (100 mg/kg) to the supplemented sows was adequately absorbed by the piglet and used metabolically for its different antioxidant functions in the body. Similar doses given to sows from gestation or during lactation have shown positive effects on the transfer and general oxidative status of the piglets [31,35,36,37].

Concerning the presence of substances in the gut-derived from the milk fermentation by microbial action or transferred by the sow, as is the case of SCFAs (fatty acids with less than six carbons) [12], their concentrations were in accordance with those observed in the literature [10]. Acetate, propionate, and butyrate are the most abundant (constituting more than 95% of the total SCFAs) [38] and are presented in a proportion of 60:20:20 (C2, C3, C4) [38]. However, in the present research, higher levels of C4 than C3 were observed. This finding agrees with the age of the animals since butyric acid mainly comes from animal fats [39] or lactic acid fermentation [40], which are important constituents of sow’s milk [20]. Moreover, it is interesting to remark that piglets from VE-supplemented sows resulted in a significant increase in the total SCFA production in piglet’s faeces when compared with non-supplemented. Previous in vitro investigations found that VE can induce the production of SCFA [16], and studies carried out on mice reported that VE intake altered the gut microbiota composition [15,41,42]. This is a relevant result since SCFAs not only constitute a source of energy for enterocytes but also may specifically activate receptors in the gut epithelium and other multiple sites such as adipose tissue, pancreatic islets, immune system, or muscles that provide multiple beneficial roles in the regulation of metabolism [39]. SCFAs can also enhance the immune response by stimulating cytokine production in the immune cells of the host [43].

It is also of interest to highlight in the present research that the most affected SCFAs in piglet’s faeces from VE-supplemented sows were iC4 and iC5. There are no further studies on the effects of VE supplementation on mothers on the SCFAs of the litter. However, it has been reported that iC4 and iC5 come from protein fermentation, mainly from the branched-chain amino acids valine and leucine, respectively [44,45]. This would indicate that those piglets from the VE-supplemented groups might have a higher transfer by the mother or activity of bacteria responsible for protein degradation and formation of these compounds. This is an interesting aspect of piglet gut health since it has been stated that iC5 (derived from bacteroides activity) may enhance mucosal immunity by promoting intestinal IgA production [46]. iC4 has also been shown to have interesting effects on intestinal health since it may serve as a fuel in colonocytes when butyrate availability is defective [47], and several studies indicate its possible role (iC4) in ionic regulation, being able to act as a regulator of Na absorption in the colon [48,49].

Also, piglets from VE-supplemented sows had the highest level of C4 in the present study when compared to the other groups. Other authors found that VE increased the abundance of Roseburia, a butyrate producer [50], which would be, in turn, the highest butyrate levels of the VE group. Liu et al. [15] also found that VE attenuated the depletion of butyrate-producing bacteria in mice with induced colitis. Butyrate has been related to interesting functions in the organism. It improves barrier function by promoting tight junction (TJ) assembly [15,51], induces the production of IL18, which regulates gut microbiota composition and antimicrobial peptides [52,53], and can improve the production of immunoglobulin A cells [54] or functionality of goblet cells [55,56], maintaining microbial homeostasis.

Contrary to what was observed for VE supplementation, HXT did not produce such marked effects on the piglet’s faecal SCFAs content. Thus, only the concentration of iC5 increased by the dietary treatment of the sows. The most evident effects of HXT supplementation seem to be directed towards a decrease in the faecal content of SCFAs. In fact, it was found that the piglets from mothers supplemented with HXT had lower levels of C4, and the lowest amounts were found in the group that received only HXT. HXT has been shown to have antioxidant effects [19,57] and protection against inflammation in the intestinal mucosa [18], as well as a regenerative effect on the barrier at this level [17]. Moreover, maternal supplementation with HXT seems also to be effective in improving the performances and growth pattern of piglets, mainly at birth, although piglets from sows that received HXT did not show changes in performances after weaning [31].

In relation to the changes in the levels of SCFAs of the HXT group associated with milk composition, it is interesting to point out that in a previous study carried out within the scope of this same research project [20], sows supplemented with HXT produced colostrum with a higher proportion of unsaturated fatty acids. The fact that the colostrum and milk of mothers supplemented with HXT had a higher proportion of PUFA makes it more susceptible to the production of substances derived from lipid oxidation, such as MDA, which would be directly related to the oxidative status of the piglet [20,31] and the SCFA-forming bacterial population. In this sense, Bozinou et al. [58] found a high correlation between microbial population and oxidative stability of food containing yogurt. Moreover, in the present study, the faecal content of SCFAs, mainly iC4, iC5, C3, or C5, was directly related to the oxidative status of the piglet or its mother. These metabolites increased linearly in a significant way with the increase in the piglets or sow’s antioxidant enzymes. Other authors suggest that there is an association between the oxidative status of the host and the intestinal microbiota along the gastrointestinal tract [2,59,60]. Despite the higher unsaturation of milk from the HXT group and the direct relationship between milk and piglets’ TBARs [20], HXT given to sows showed a similar positive effect as VE for reducing piglet’s plasma TBAR production post-weaning [31].

In view of these different effects between VE and HXT, mainly on the fecal content of SCFAs, an additive effect from the administration of both compounds would not be expected. However, although the combination of both antioxidants did not modify the gut total SCFAs nor the majority compounds, iC4 and iC5 significantly increased. This could be due to different oxidative ranges at the intestine or changes in the composition of colostrum or milk throughout lactation since the VE+HXT sows were the ones that produced the day-7 milk with the lowest PUFA content, although day-20 milk had the highest content of this fatty acid [20].

To quantify to what extent the changes produced in the milk composition by the administration of the VE or HXT to the mother could affect the piglet’s SCFAs, correlations and regression equations were carried out. The SCFAs most correlated with colostrum or milk composition were iC4, iC5, and C4. Even though the stool samples were collected after weaning, it seems that colostrum intake during the first hours of lactation and its composition seems crucial to establish differences in the intestinal environment [61], as well as developmental changes in intestinal function [62]. Thus, C4 levels were inversely and significantly related to PUFA (mainly C18) of colostrum. It is also interesting to remark on the high correlation and linear and positive relationship observed between colostrum C20:2, C20:3, and C20:4 n-6 and the level of iC4 and iC5. The C20:2 fatty acid is the main source of C20:3 n-6 (immediate precursor of prostaglandins and thromboxanes) by alternative use of Δ-8-desaturase, whereas C20:3 n-6 is the immediate precursor of C20:4 n-6 (arachidonic acid, main component of the phospholipids membranes) [63]. Although n-6 unsaturated fatty acids seem to have a negative effect on the adhesion of certain Lactobacillus, arachidonic acid promotes growth and mucus adhesion of *L. casei* [64]. Therefore, a greater proportion of these fatty acids in the colostrum, between other components, might facilitate the maturation of the intestinal epithelium, establishment of the microbial population, and greater functionality.

Similar results were observed between day-7 milk composition and faecal C5 content. Moreover, the proportion of C5, iC5, and iC4 were positive and linearly affected by monounsaturated fatty acids (MUFA), desaturase capacity of the sow, and inversely by saturated fatty acids. Also, a direct and positive relationship was observed between total MUFA and total SCFAs. Tsutsumi et al. [65] reported that long-chain MUFA fatty acids improved endothelial function by altering the gut microbial environment, and that stimulated the production of SCFAs. Other authors [66] observed that vitamin E was associated with a decrease in harmful microbial populations. There is hardly any information relating the different types of fat or their composition to the production of SCFAs or the intestinal environment. The results of the present study reinforce the idea that the relationship observed between MUFA fatty acids and greater production of SCFAs would be indirectly associated with the antioxidant effect of VE on desaturases, which are enzymes highly sensitive to oxidation [67]. Thus, while the composition of the milk explained approximately between 20–40% of the variation in the content of SCFAs, the plasmatic levels of VE explained at least 45%, as observed in the regression equations of the present study.

Moreover, the positive effect of dietary VE associated with changes in colostrum or milk composition was observed on the intestinal morphology. Thus, those colostrum/milk fatty acids produced by VE supplementation (higher MUFA proportion, desaturase indices, or VE concentration) [20] were directly related to enhanced villi number and crypt depth, which would represent an increase in the mass of the intestine. Other authors [68] reported that colostrum intake and its composition may enhance duodenal villus size and reduce epithelial cell proliferation. Since crypts move upwards to form the villi, a lower cell regeneration rate (apoptosis), higher crypt depth, and longer villi would lead to higher mucosal surface and nutrient absorption [69]. In this sense, a negative correlation was observed between C18-PUFA (mainly C18:2 n-6, which was higher in day-20 milk from HXT sows) and villi height. PUFA-enriched diets might negatively affect gut morphology [70], whereas positive effects have been observed with the use of diets with MUFA-enriched antioxidants [71]. In addition, high correlations were found between the oxidative status of the sow and the number of goblet cells in the intestine of the piglet. It is unknown how the goblet cells regulate mucus production, but it has been suggested that mucus secretion depends on the intestinal endoplasmic reticulum stress [72]. Thus, in the present research, there is an inverse relationship between the number of goblet cells and milk from VE-supplemented sows, which did provide a lesser amount of TBARs to the piglet. The effect of VE-supplementation on goblet number could also be related to the higher C4 concentrations produced by this group since there has been an observed decrease in these producing-mucus cells by SCFA infusion [56]. Moreover, sodium butyrate administration has been associated with a thinner small intestinal mucosa [73] and crypt depth [74], as observed in the present study in piglets from the VE-supplemented group, which could result in different nutrient absorption.

In addition, the total fatty acid profile of faecal samples was also evaluated in the present research as indicators of fatty acid absorption, possible inflammatory status [24], or microbial synthesis activity at the intestinal level. There were very slightly significant changes, which would indicate adequate absorption in the intestine. However, HXT groups tended to have a higher C20:4 to C20:2 ratio, higher C22:6 n-3 (DHA), and lower elongase C14-C16 in faeces, which would be related to worse inflammatory status and gut health [24,63]. Specifically, it has been observed that DHA has a high impact on tight junction protein and on the modulation of epithelial permeability under physiological conditions of inflammatory stress [75]. Lower incorporation of this fatty acid in the intestinal phospholipid membrane (due to the greater losses in faeces) in the HXT group could indicate certain alterations in functionality at this level.

The less favorable intestinal environment in piglets from HXT-supplemented sows was confirmed from the analysis of the gene expression of tight junction protein. These animals presented a significantly lower expression of *OCLN*, which coincides with other authors who evaluated chronic intestinal processes when compared to the control [76,77]. *OCLN* expression could be inhibited by interkeukin-1 in the presence of inflammation [77]. Thus, high dietary n-6 may decrease the presence of *OCLN* in tight junction complexes [75]. Moreover, these results agree with the positive relationship between butyrate and *OCLN* gene expression described in in vitro studies [78], as butyric acid is reduced by HXT supplementation according to our results.

These results also coincide with the relationship observed between some specific faecal fatty acids and the SCFAs. Thus, a higher proportion of PUFA in faeces (as observed in HXT groups) would be related to lower SCFA production, mainly C4. However, others, especially C16:0 (hexadecanoic acid) or saturated fatty acids that can be obtained from the fermentation of C2 or C4 [79,80], showed the highest positive correlations with the SCFAs content since they are related to microbial activity [24,79,81]. The faecal C16:0 has also been inversely related to the degree of disease in patients with intestinal chronic inflammation [81]. Taking into account that the piglets from VE-supplemented sows had the highest content of C4, this could be transformed into C16:0 by microbial action, creating a more favorable environment and a greater production of SCFAs and intestinal health. Therefore, according to the results of the present study, the level of C18-polyunsaturated fatty acids of the colostrum could determine changes in the membrane incorporation of specific fatty acids, an initial microbial population, and C4 production and, therefore, different activity in regulating tight junctions of membrane and intestinal health.

Finally, the relationships between the piglet’s faeces composition and their growth were quantified. The relationship between the production of SCFAs and growth was hardly evident, taking into account that these constitute a small contribution of energy. However, other authors found that changes in gut microbiota and derived-metabolite formation resulted in increases in some organs and body weight [41,82]. On the contrary, a significant inverse relationship between the faecal percentage of DHA (which tended to increase in the HXT group) and the size of the piglets were observed. A greater presence of DHA in faeces could be related to lower incorporation of it into the gut membrane, where it performs its main function, with negative consequences on nutrient absorption and its anti-inflammatory state [75].

## 5. Conclusions

In conclusion, the administration of VE to sows from late gestation produces an increase in piglet’s SCFAs concentration (mainly butyric acid) in faeces when compared with those from non-supplemented sows. Piglet’s SCFAs were mainly influenced by the circulating vitamin E or piglets’ and sows’ oxidative status, as well as by colostrum or milk composition. The faecal fatty acid profile and lesser SCFA production in piglets from HXT-supplemented sows would indicate the superiority of VE over HXT on the intestinal environment and, consequently, on the piglets’ gut health.

## Figures and Tables

**Table 1:** Characteristics and α-tocopherol concentration of piglets’ faeces from sows given α-tocopheryl acetate (VE: 30 vs. 100 mg/kg) or hydroxytyrosol (HXT: 0 vs. 1.5 mg/kg) from day 85 of gestation.

	Control ^1^	VE ^2^	HXT ^3^	VE+HXT ^4^	VE-30	VE-100	HXT-0	HXT-1.5	RMSE ^5^	*p* VE ^6^	*p* HXT	*p* VE X HXT
***Faecal characteristics***												
Moisture, %	79.42	79.43	79.22	78.56	79.32	79.00	79.42	78.89	3.306	0.7381	0.5774	0.7281
Fat, %	18.90	16.72	18.52	17.72	18.71	17.22	17.81	18.12	4.255	0.2327	0.8026	0.5767
Fat, % DM ^7^	3.86	3.48	3.82	3.85	3.84	3.66	3.67	3.83	1.073	0.5664	0.6004	0.5113
α-tocopherol, µg/g	201.00	263.38	219.82	178.66	210.41	221.02	232.19	199.24	102.788	0.7225	0.2728	0.0880

^1^ Control = 30 mg of α-tocopheryl acetate/kg feed + 0 mg/kg hydroxytyrosol; ^2^ VE = 100 mg of α-tocopheryl acetate/kg feed + 0 mg/kg hydroxytyrosol; ^3^ HXT = 30 mg of α-tocopheryl acetate/kg feed + 1.5 mg/kg hydroxytyrosol; ^4^ VE+HXT = 100 mg of α-tocopheryl acetate/kg feed + 1.5 mg/kg hydroxytyrosol; ^5^ RMSE = Root mean square error (pooled SD); ^6^ *p* = differences were statistically different when *p* < 0.05; ^7^ DM = dry matter.

**Table 2:** Faecal SCFAs composition (mg/g) of piglets from sows given α-tocopherol (VE: 30 vs. 100 mg/kg) or hydroxytyrosol (HXT: 0 vs. 1.5 mg/kg) from day 85 of gestation.

	Control ^1^	VE ^2^	HXT ^3^	VE+HXT ^4^	VE-30	VE-100	HXT-0	HXT-1.5	RMSE ^5^	*p* VE ^6^	*p* HXT	*p* VE X HXT
***Piglet’s SCFAs***												
Acetic acid	0.69	0.61	0.50	0.66	0.59	0.63	0.65	0.58	0.275	0.636	0.427	0.172
Propionic acid	0.28	0.23	0.21	0.33	0.25	0.28	0.26	0.27	0.148	0.504	0.801	0.092
Isobutyric acid	0.03 ^b^	0.03 ^b^	0.02 ^b^	0.05 ^a^	0.02 ^b^	0.04 ^a^	0.03	0.04	0.019	0.007	0.189	0.017
Butyric acid	0.67	0.94	0.16	0.59	0.42 ^b^	0.76 ^a^	0.80 ^A^	0.37 ^B^	0.536	0.050	0.018	0.678
Isovaleric acid	0.05 ^b^	0.06 ^b^	0.04 ^b^	0.12 ^a^	0.04 ^b^	0.09 ^a^	0.05	0.08	0.044	0.003	0.090	0.012
Valeric acid	0.04	0.06	0.03	0.05	0.04	0.06	0.05	0.04	0.033	0.079	0.437	0.699
Caproic acid	0.01	0.02	0.01	0.01	0.01	0.02	0.01	0.01	0.010	0.871	0.510	0.188
∑SCFAs ^7^	1.76	1.94	0.88	1.76	1.32 ^b^	1.85 ^a^	1.85 ^A^	1.32 ^B^	0.711	0.022	0.022	0.122
∑C2+C3+C4 ^8^	1.64	1.79	0.79	1.51	1.21 ^b^	1.65 ^a^	1.71 ^A^	1.15 ^B^	0.677	0.046	0.012	0.179
∑iC4+iC5 ^9^	0.07 ^b^	0.08 ^b^	0.06 ^b^	0.17 ^a^	0.06 ^b^	0.13 ^a^	0.08	0.11	0.061	0.003	0.096	0.014

^1^ Control = 30 mg of α-tocopheryl acetate/kg feed + 0 mg/kg hydroxytyrosol; ^2^ VE = 100 mg of α-tocopheryl acetate/kg feed + 0 mg/kg hydroxytyrosol; ^3^ HXT = 30 mg of α-tocopheryl acetate/kg feed + 1.5 mg/kg hydroxytyrosol; ^4^ VE+HXT = 100 mg of α-tocopheryl acetate/kg feed + 1.5 mg/kg hydroxytyrosol; ^5^ RMSE = Root mean square error (pooled SD); ^6^ *p* = differences were statistically different when *p* < 0.05; ^7^ ∑SCFAs = sum of total short chain fatty acids; ^8^ ∑C2+C3+C4 = sum of acetic acid + propionic acid + butyric acid; ^9^ ∑iC4+iC5 = sum of isobutyric acid + isovaleric acid; ^a,b,A,B^ Letter with different superscript were statistically significant.

**Table 3:** Pearson correlation coefficients (r) and significant regression equations between oxidative status and faecal SCFAs of post-weaning piglets from sows given α-tocopheryl acetate (VE: 30 vs. 100 mg/kg) or hydroxytyrosol (HXT: 0 vs. 1.5 mg/kg) from day 85 of gestation.

Variable Y	Intercept s.d. ^1^	Slope s.d.	Variable X	r	R^2^	*p* Linear ^2^
***Piglet’s faecal SCFAs* (mg/g)**			***piglet’s oxidative status post-weaning***			
Propionic acid (C3)	0.128 ± 0.52	0.376 ± 0.15	SOD, U/mL^5^	0.44	0.19	0.022
Propionic acid (C3)	0.127 ± 0.05	0.069 ± 0.03	GSH, µM ^6^	0.42	0.18	0.033
Isobutyric acid (iC4)	−0.008 ± 0.01	0.031 ± 0.01	plasma α-tocopherol, µg/mL	0.70	0.49	0.000
Isovaleric acid (iC5)	−0.029 ± 0.02	0.076 ± 0.02	plasma α-tocopherol, µg/mL	0.69	0.47	0.000
∑IC4+IC5 ^3^	−0.038 ± 0.03	0.108 ± 0.02	plasma α-tocopherol, µg/mL	0.70	0.48	0.000
∑SCFAs ^4^	0.710 ± 0.44	0.721 ± 0.30	plasma α-tocopherol, µg/mL	0.44	0.19	0.024
			***sow’s plasma oxidative status at day 20***			
Propionic acid (C3)	0.161 ± 0.04	0.062 ± 0.02	plasma GSH/GSSG ^7^	0.51	0.25	0.005
Valeric acid (C5)	0.025 ± 0.01	0.011 ± 0.00	plasma GSH/GSSG	0.45	0.20	0.016

^1^ s.d. = standard deviation of mean; ^2^ *p* = differences were statistically different when *p* < 0.05; ^3^ ∑iC4+iC5; ^4^ ∑SCFAs = sum of total short chain fatty acids; ^5^ SOD = superoxide dismutase; ^6^ GSH = total glutathione; ^7^ GSH/GSSG = reduced/oxidised glutathione.

**Table 4:** Pearson correlation coefficients (r) and significant regression equations between colostrum composition and faecal SCFAs of post-weaning piglets born from sows given α-tocopheryl acetate (VE: 30 vs. 100 mg/kg) or hydroxytyrosol (HXT: 0 vs. 1.5 mg/kg) from day 85 of gestation.

Variable Y	Intercept s.d. ^1^	Slope s.d.	Variable X	r	R^2^	*p* Linear ^2^
***Faecal SCFAs* (mg/g)**			***Colostrum***			
Butyric acid (C4)	2.023 ± 0.749	−0.099 ± 0.047	C18:2 n-6	−0.41	0.17	0.045
	2.134 ± 0.809	−0.096 ± 0.046	∑PUFA ^3^	−0.41	0.17	0.047
Isobutyric acid (iC4)	−0.031 ± 0.015	0.110 ± 0.031	C20:2	0.62	0.38	0.002
	−0.024 ± 0.017	0.208 ± 0.076	C20:3 n-6	0.51	0.26	0.013
	−0.023 ± 0.016	0.039 ± 0.013	C20:4 n-6	0.54	0.29	0.008
Isovaleric acid (iC5)	−0.100 ± 0.036	0.298 ± 0.073	C20:2	0.67	0.44	0.001
	−0.079 ± 0.042	0.546 ± 0.187	C20:3 n-6	0.54	0.29	0.008
	−0.081 ± 0.037	0.109 ± 0.031	C20:4 n-6	0.60	0.36	0.003
	−0.003 ± 0.012	3.639 ± 0.865	Elongase C18-C20	0.68	0.46	0.000

^1^ s.d. = standard deviation of mean; ^2^ *p* = differences were statistically different when *p* < 0.05; ^3^ ∑PUFA = sum of polyunsaturated fatty acids.

**Table 5:** Pearson correlation coefficients (r) and significant regression equations between 7 and 20-milk composition and SCFAs of post-weaning piglets born from sows given α-tocopheryl acetate (VE: 30 vs. 100 mg/kg) or hydroxytyrosol (HXT: 0 vs. 1.5 mg/kg) from day 85 of gestation.

Variable Y	Slope s.d. ^1^	Slope s.d.	Variable X	r	R^2^	*p* Linear ^2^
***Faecal SCFAs* (mg/g)**			***Day-7 milk***			
Isobutyric acid (iC4)	−0.066 ± 0.031	0.003 ± 0.001	C18:1 n-9	0.53	0.28	0.007
	0.075 ± 0.023	−0.001 ± 0.001	∑SAT ^3^	−0.44	0.20	0.034
	−0.114 ± 0.041	0.003 ± 0.001	∑MUFA ^4^	0.57	0.33	0.003
	−0.106 ± 0.045	0.241 ± 0.083	Δ-9-desaturase	0.52	0.27	0.008
Isovaleric acid (iC5)	−0.090 ± 0.048	0.004 ± 0.001	C18:1 n-9	0.52	0.27	0.011
	−0.288 ± 0.111	0.007 ± 0.002	∑MUFA	0.54	0.29	0.005
	−0.141 ± 0.072	0.337 ± 0.132	Δ-9-desaturase ^5^	0.49	0.24	0.019
Valeric acid (C5)	0.256 ± 0.052	−0.007 ± 0.002	C16:0	−0.65	0.42	0.001
	0.002 ± 0.016	0.094 ± 0.032	C16:1 n-9	0.53	0.28	0.007
	−0.176 ± 0.062	0.006 ± 0.002	C18:1 n-9	0.60	0.36	0.002
	−0.023 ± 0.020	0.190 ± 0.054	C20:1 n-9	0.59	0.35	0.002
	−0.006 ± 0.024	0.257 ± 0.058	**C20:2**	0.68	0.46	0.000
	−0.007 ± 0.013	0.265 ± 0.079	C20:3 n-6	0.59	0.35	0.003
	−0.008 ± 0.024	0.079 ± 0.035	C20:4 n-6	0.42	0.18	0.036
	0.277 ± 0.066	−0.006 ± 0.002	∑SAT	−0.60	0.36	0.002
	−0.203 ± 0.094	0.005 ± 0.002	∑MUFA	0.48	0.23	0.015
	−0.257 ± 0.093	0.550 ± 0.171	Δ-9-desaturase	0.56	0.31	0.004
∑SCFAs ^6^	−4.102 ± 2.181	0.118 ± 0.046	∑MUFA	0.46	0.21	0.017
			***Day-20 milk***			
Valeric acid (C5)	−0.009 ± 0.023	0.095 ± 0.041	C16:1n-9	0.45	0.20	0.032
	0.149 ± 0.040	−0.121 ± 0.042	∑n-3 ^7^	−0.54	0.29	0.010

^1^ s.d. = Standard deviation of mean; ^2^ *p* = differences were statistically different when *p* < 0.05; ^3^ ∑SAT = Sum of total saturated fatty acids; ^4^ ∑MUFA = Sum of total monounsaturated fatty acids; ^5^ Δ-9—desaturase index = (C14:1 + C16:1 + C18:1)/C14:0 + C14:1 + C16:0 + C16:1 + C18:0 + C18:1); ^6^ ∑SCFAs = sum of total short chain fatty acids; ^7^ ∑n-3 = Sum of total n-3 fatty acids.

**Table 6:** Pearson correlation coefficients (r) and significant regression equations between piglet’s intestine morphology (post-weaning) and oxidative status or colostrum or milk composition from their mother given α-tocopheryl acetate (VE: 30 vs. 100 mg/kg) or hydroxytyrosol (HXT: 0 vs. 1.5 mg/kg) from day 85 of gestation.

Variable Y	Intercept s.d. ^1^	Slope s.d.	Variable X	r	R^2^	*p* Linear ^2^
***Piglet’s intestine morphology***			***Sow’s oxidative status***			
Goblet cells number	10.95 ± 3.1	6.01 ± 2.8	Sow’s plasma GSSG, µM ^3^	0.40	0.16	0.041
Goblet cells number	14.29 ± 1.8	26.45 ± 12.6	Sow’s plasma MDA, mM ^4^	0.39	0.15	0.047
			***Colostrum composition***			
Villi number	3.28 ± 1.1	0.67 ± 0.3	C16:1 n−7	0.46	0.21	0.031
	10.55 ± 1.6	−0.78 ± 0.3	C18:0	−0.56	0.32	0.007
	15.97 ± 4.1	−0.33 ± 0.1	∑SAT ^5^	−0.49	0.24	0.022
	1.89 ± 1.4	0.57 ± 0.2	C18:1/C18:0	0.53	0.28	0.011
Crypt depth, µm	126.53 ± 105.2	3714.46 ± 1422.3	∆5+∆6 ^6^	0.50	0.25	0.017
			***Day-20 milk composition***			
Villi height, µm	693.50 ± 153.5	−33.57 ± 14.1	C18:2 n−6	−0.48	0.23	0.028
	707.19 ± 157.5	−31.90 ± 13.2	∑n−6−PUFA ^7^	−0.48	0.23	0.026
Crypt depth, µm	303.20 ± 41.0	91.62 ± 38.1	α−tocopherol	0.48	0.23	0.027
	188.03 ± 78.2	461.69 ± 172.3	C20:1 n−9	0.52	0.27	0.015
Villi number	9.20 ± 1.52	−0.66 ± 0.27	C18:0	−0.49	0.25	0.026
Goblet cells number	22.91 ± 2.5	−5.34 ± 2.3	α−tocopherol	−0.48	0.23	0.032

^1^ s.d. = Standard deviation of mean; ^2^ *p* = differences were statistically different when *p* < 0.05; ^3^ GSSG = oxidized form of Glutathione; ^4^ MDA = malonhyaldehyde; ^5^ ∑SAT = Sum of total saturated fatty acids; ^6^ Δ-5+Δ-6 − desaturase index = (C18:3n-6 + C18:4n-3 + C20:4n-6)/(C18:2n-6 + C18:3n-3 + C18:3n-6 + C18:4n-3 + C20:4n-6 + C20:3n-6); ^7^ ∑n-6-PUFA = Sum of total n-6 fatty acids.

## Data Availability

Data are contained within the article.

## Appendix A

**Table A1:** Analysed composition and fatty acid proportion of the experimental diets.

Analysed Composition ^1^	CONTROL	VE ^2^	HXT	HXT+VE
Dry matter, %	90.8	89.8	91.1	91.2
Crude Protein, %	13.1	14.0	15.0	14.5
Fat, %	4.3	3.6	3.8	3.7
Ash, %	6.5	6.4	6.9	6.0
Fiber, %	4.3	4.4	4.3	4.4
Starch,%	49.0	46.1	41.1	43.0
				
Vitamin E, mg/kg	70.5	103.6	65.4	114.1
***Fatty acid composition***				
C14:0	0.60	0.66	0.56	0.60
C16:0	19.80	21.48	19.10	20.11
C16:1n-9	0.12	0.15	0.12	0.12
C16:1n-7	0.79	0.87	0.73	0.78
C18:0	4.77	4.80	4.45	4.54
C18:1n-9	28.97	25.26	30.33	26.94
C18:1n-7	1.92	1.49	1.57	1.60
C18:2n-6	38.97	40.81	38.97	40.89
C18:3n-3	3.04	3.44	3.15	3.36
C20:0	0.30	0.26	0.30	0.31
C20:1n-9	0.52	0.58	0.55	0.58
∑SAT	25.47	27.20	24.41	25.55
∑MUFA	32.33	28.35	33.29	30.02
∑PUFA	42.01	44.25	42.11	44.25

^1^ Ingredients (%): corn: 16.03; wheat: 10; Barley: 50; soya meal 47: 6.73; wheat bran: 6.54; pork lard: 1; beetroot pulp: 5; calcium carbonate: 1.98; bicalcium phosphate: 0.54; salt: 0.5; L-lysine: 0.50; Methionine: 0.04; threonine: 0.18; choline chloride: 0.03; premix: 0.3 (per kg/diet: vitamin A: 12,000 IU; Vitamin D_3_: 1400 IU; Vitamin B_1_: 1.1 mg; Vitamin B_2_: 6 mg; Vitamin B_12_: 0.08 mg; Vitamin B_6_: 12 mg; Nicotinic acid: 21 mg; Biotin: 0.12 mg; Pantothenic acid: 12 mg; Vitamin K_3_: 1.1 mg; Choline chloride: 225 mg; Fe (ferrous carbonate): 60 mg; Cu (pentahydrate sulphate): 14.2 mg; Zn (oxide): 100 mg; Mn (monohydrate sulphate): 30 mg; I (potasium iodure): 0.8 mg; Se (sodium selenite): 0.3 mg. ^2^ Calculated metabolizable energy (ME) based on ingredient composition = 3030 Kcal ME/kg.

**Table A2:** Primer design for qPCR.

Gene Symbol	Gene Name	GenBank ID	Forward Primer Sequence	Reverse Primer Sequence
***OCLN***	Occludin	NM_001163647	CTCGTCCAACGGGAAAGTGA	CGTGTAGTCTGTCTCGTAATGGT
***ZO1***	Zonula Occludens 1	AJ318101	CTGGGCTCTTGGCTTGCTAT	GGCTAACTGCTCAGCTCTGT
***GADPH***	Glyceraldehyde-3-phosphate dehydrogenase	AF069649	TGGTGAAGGTCGGAGTGAAC	GAAGGGGTCATTGATGGCGA
***EEF2***	Eukaryotic translation elongation factor 2	XM_003354002	CGACACTTTGAGACTGTCCAGACT	AAGTGGGCCCCAGAAACC
